# Cardiac Sarcoidosis or Giant Cell Myocarditis? On Treatment Improvement of Fulminant Myocarditis as Demonstrated by Cardiovascular Magnetic Resonance Imaging

**DOI:** 10.1155/2012/647041

**Published:** 2011-11-15

**Authors:** Hari Bogabathina, Peter Olson, Vikas K. Rathi, Robert W. W. Biederman

**Affiliations:** ^1^Department of Cardiovascular Magnetic Resonance Imaging, Gerald McGinnis Cardiovascular Institute, Allegheny General Hospital, 320 East North Avenue, Pittsburgh, PA 15212, USA; ^2^Department of Cardiovascular Magnetic Resonance, Bon Secours Heart & Vascular Institute, 5949 Harbour Park Drive, Midlothian, VA, USA

## Abstract

Giant cell myocarditis, but not cardiac sarcoidosis, is known to cause fulminant myocarditis resulting in severe heart failure. However, giant cell myocarditis and cardiac sarcoidosis are pathologically similar, and attempts at pathological differentiation between the two remain difficult. We are presenting a case of fulminant myocarditis that has pathological features suggestive of cardiac sarcoidosis, but clinically mimicking giant cell myocarditis. This patient was treated with cyclosporine and prednisone and recovered well. This case we believe challenges our current understanding of these intertwined conditions. By obtaining a sense of severity of cardiac involvement via delayed hyperenhancement of cardiac magnetic resonance imaging, we were more inclined to treat this patient as giant cell myocarditis with cyclosporine. This resulted in excellent improvement of patient's cardiac function as shown by delayed hyperenhancement images, early perfusion images, and SSFP videos.

## 1. Introduction

Fulminant myocarditis is an extremely severe form of heart failure, with short duration of onset, requiring the use of inotropic support and often cardiac mechanical assistive support. Giant cell myocarditis (GCM) is a well-noted cause of fulminant myocarditis [1]. Infiltrative cardiomyopathies, including idiopathic granulomatous myocarditis and cardiac sarcoidosis (CS), typically run a chronic course and are not known to cause fulminant myocarditis [1]. Recently, fulminant myocarditis has been designated a Class 1 indication for endomyocardial biopsy (EMB), as patients with definitive pathological diagnosis and appropriate treatment have good prognosis [1].

There is an ongoing debate whether GCM is a distinct pathological entity from CS or GCM is a part of the spectrum of pathology in CS [2]. The multicenter observational study by Okura et al. has given some insight into the pathological and clinical distinctions between GCM and CS. GCM pathologically has giant cells, eosinophils, lymphocytic inflammatory infiltrate, and prominent myocyte necrosis [2, 3]. CS on the other hand has noncaseating granulomatous inflammation, predominant fibrosis, and without prominent myocyte necrosis [2]. Notably, both GCM and CS had equivalent numbers of giant cells [2]. CS had a more indolent clinical course, with more likelihood of bradyarrhythmias, and better prognosis [2].

Until recently, it has been difficult to diagnose CS premortem. Endomyocardial biopsy although definitive when positive has a poor negative predictive value secondary to skip lesions [4]. Recently, DHE-CMR has shown promise in reliably diagnosing CS with excellent sensitivity and specificity. In our lab, we diagnose CS definitively by utilizing DHE-CMR [5]. Of the 81 patients with biopsy-proven extracardiac sarcoidosis 21 patients (26%) had CS by DHE-CMR [6]. DHE-CMR demonstrated sensitivity, specificity, positive predictive value, negative predictive value, and overall accuracy of 100%, 78%, 55%, 100%, and 83%, respectively, in the diagnosis of CS among patients with pulmonary sarcoidosis [7]. To our knowledge, there has only been two case reports of GCM diagnosed by CMR [8, 9]. We describe a case of fulminant myocarditis, which has pathological features of CS, clinically mimicking GCM, treated as GCM based on the extensive myocardial involvement on CMR.

## 2. Case Presentation

A 42-year-old African-American male presented to our hospital with a 2-week history of rapidly progressive exertional dyspnea. He had no significant past medical history, did not take any medications, had no allergies, occasional cigarette smoking, drank an average of six-pack of beer a week, and his parents had hypertension and diabetes.

Cardiac enzymes were negative, BNP level was 843, and D-dimer was elevated at 3.7. A urine drug screen, HIV 1/HIV 2 antibodies, and hepatitis viral panel were negative. Rheumatoid factor was normal, ANA titer was mildly positive with a speckled pattern, angiotensin converting enzyme level was high at 70 U/L (normal 7 to 46), histoplasma antigen was negative, parvovirus B-19 IgM antibody was negative, and parvovirus IgG antibody was positive suggesting a past infection. Patient's EKG showed sinus rhythm, low voltage, and poor R-waves in inferior and anterior leads. CXR showed pulmonary edema and cardiomegaly, with nodular pattern in the periphery. CT chest with contrast showed no evidence of pulmonary embolism, significant degree of mediastinal, hilar, right peridiaphragmatic lymphadenopathy, small pleural effusion, small pericardial effusion, and bilateral multifocal consolidation.

TTE showed severe global hypokinesis with regional variation, mostly sparing the apex.

Tc-99 tetrofosmin with adenosine stress testing showed a medium to large, severe, fixed anterior perfusion defect with sparing of the apex, suggesting an anterior infarct, although the distribution was atypical for a proximal LAD occlusion. Severe global LV systolic dysfunction was also noted.

Left and right heart catheterization showed normal coronary arteries, elevated right atrial pressures, moderate pulmonary hypertension, elevated pulmonary capillary wedge pressure, elevated LVEDP, decreased cardiac output and index, no intra- or extracardiac shunts were noted. Patient was anticoagulated and placed on intra-aortic balloon pump for afterload reduction.

**Figure 1:**
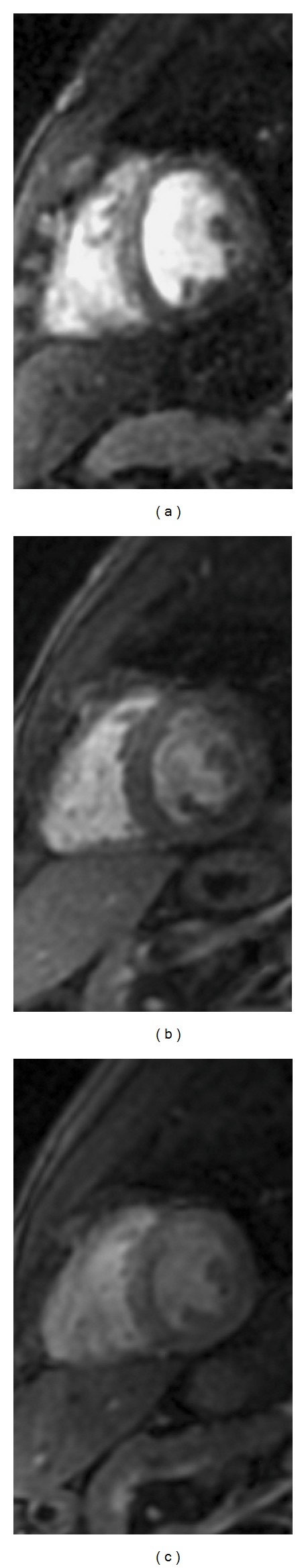
First-pass perfusion images. Subendocardial perfusion defects seen on these first-pass perfusion images in the initial exam when patient was critically ill (a) do not conform to any coronary distribution and have improved in the study done in seven weeks on cyclosporine and prednisone (b), and show stabilization from then on as evidenced on the study done in nine months (c).

**Figure 2:**
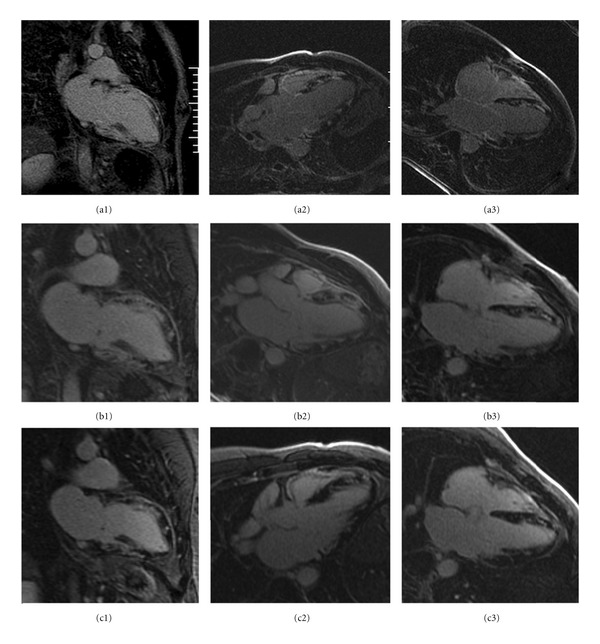
Delayed gadolinium hyperenhancement images. DHE-CMR pattern of patchy delayed hyperenhancement secondary to giant cell myocarditis or a severe form of cardiac sarcoidosis. Patchy hyperenhancement in left ventricular wall and diffuse enhancement in right ventricular wall in the initial exam (a1, a2, and a3) has improved to coalescence of hyperenhancement in seven weeks on cyclosporine and prednisone (b1, b2, and b3). The nine-month exam (c1, c2, and c3) shows a stabilization of change in hyperenhancement as compared to the seven-week exam.

Cardiovascular magnetic resonance imaging (CMR) was done for further evaluation of fulminant myocarditis. CMR showed top normal left ventricular size, mildly dilated by 3D, LVEF of 27%, with severe regional wall dysfunction, worse at base and sparing the apex (see Video 1 in Supplementary Material available on line at doi:10.1155/2011/647041). First-pass perfusion demonstrated nonspecific subtle subendocardial hypoperfusion defect, not following any coronary distribution (Figure 1). Delayed hyperenhancement imaging (DHE) showed an extremely heterogeneous, dense, and patchy, near complete enhancement of the myocardium, with increased T1 signal by gadolinium imaging. Late imaging after the postedema washout period reveals a marked patchy signal consistent with a severe inflammatory or infiltrative process (Figure 2). Right ventricular segmental dysfunction exactly colocalized with the transmural RV signal. Right paratracheal and perihilar lymphadenopathy was also noted. The official CMR report suggested that this pattern was most consistent with GCM.

**Figure 3:**
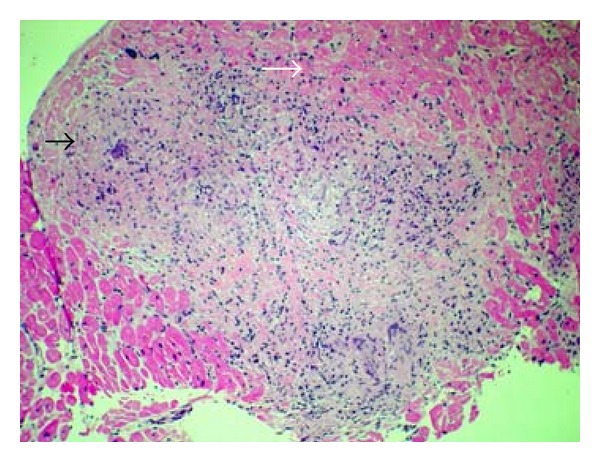
Hematoxylin and Eosin staining of myocardial biopsy specimen at 100x resolution demonstrating well-formed granuloma (black arrow) and myocardial fascicles (white arrow).

**Figure 4:**
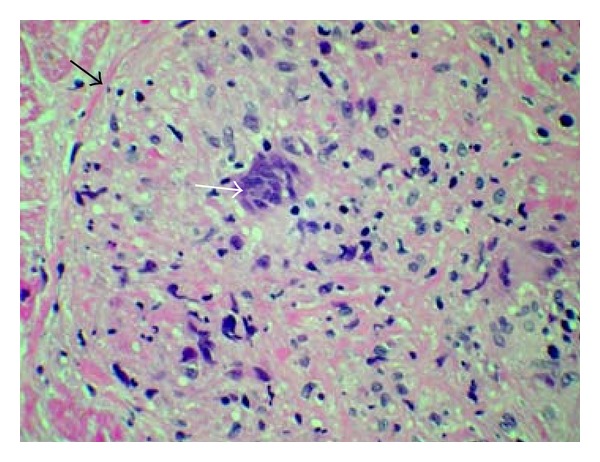
Hematoxylin and Eosin staining of myocardial biopsy specimen at 200x resolution demonstrating well-formed granuloma (black arrow) with a multinucleated giant cell in the center (white arrow).

Based on the CMR findings and the clinical presentation EMB was done. The EMB showed noncaseating granulomatous inflammation with many multinucleated giant cells, histiocytes, and mild chronic inflammation (Figures 3 and 4). Special stains were negative for organisms. No viral cytopathic effect was seen. Iron stain was negative. Thioflavin-T stain and congo red stain for amyloid were negative. Sarcoidosis/granulomatous myocarditis was favored over idiopathic giant cell myocarditis because of the presence of well-formed granulomas and granulomatous inflammation. Interestingly, the first official pathology read was signed out as GCM. Thus, the patient was initiated on prednisone and cyclosporine 50 mg PO BID which was later increased to 75 mg PO BID in 6 days, in specific treatment of GCM.

With rapid clinical improvement from CHF, the IABP was removed. After clinical stabilization, the patient was discharged home on carvedilol, aspirin, lisinopril, prednisone slow taper over 5 weeks, cyclosporine at 75 mg PO BID, and Bactrim SS for prophylaxis while on cyclosporine.

Repeat CMR 7 weeks after the initial CMR showed improved LVEF to 40% (Video 2). The first-pass perfusion imaging showed hypoperfused subendocardial defects remained generalized (Figure 1). DHE showed moderate improvement and coalescence of previously seen LV myocardial enhancement with mild to moderate improvement in RV signal as well (Figure 2). Also noted was the interval improvement in the mediastinal lymphadenopathy.

Repeat CMR 9 months later showed the LVEF stabilized at 40% and RVEF at 45% (Video 3). First-pass perfusion by gadolinium revealed an unchanged generalized subendocardial, near circumferential hypoperfusion defect (Figure 1). The DHE pattern had mildly improved as well (Figure 2). Also noted was the interval improvement of the pulmonary sarcoidosis pattern since the second CMR.

## 3. Discussion

Fulminant myocarditis has a limited differential diagnosis including GCM, necrotizing eosinophilic myocarditis, lymphocytic myocarditis, and acute myocardial infarction [1]. GCM is the leading offender among the nonischemic presentations. Although, after extensive work of gathering all available cases, clinical and pathological distinctions between GCM and CS were made by Okura et al., demonstrating a significant number of cases, there is significant overlap in both pathological and clinical findings [2].

Our patient presented with severe heart failure, which quickly deteriorated into fulminant heart failure, requiring IABP and inotropic support. An intrepid diagnosis of GCM was made on CMR. EMB showed giant cells and granulomatous inflammation, pointing to a diagnosis of GCM versus CS. However, the acute clinical presentation, the extensive nature of myocarditis, involving entire heart by CMR forced us to consider a diagnosis of GCM and overlap with CS, thus treating the patient with cyclosporine and steroids. Over the next 7 weeks, patient's EF improved from 27% to 40%. Cyclosporine-based immunosuppression as treatment for GCM has been studied and showed good prognosis and prolonged transplant-free survival [10, 11]. Steroids are the mainstay of treatment for CS, and only a few studies have looked at immunosuppressive agent use for treatment [12].

Serial CMRs over a nine month period demonstrated improving ejection fraction, decreasing inflammation, and coalescing of enhancement via DHE corresponding to the changing pattern of fibrosis that correlated with marked and durable clinical improvement.

From our review of literature, there has been one reported case of GCM imaged by CMR, but diagnosis was confirmed only on pathology of explanted heart. Ongoing controversy regarding partial overlap of GCM with CS suggests that immunomodulatory therapies are beneficial in both settings, such that astute clinical recognition and rapid institution of immunologic therapy are critical to patient prognosis.

## 4. Conclusion

The extent of cardiac involvement and improvement on immunomodulator and steroid treatment is well demonstrated by CMR imaging. Perfusion CMR imaging and DHE-CMR imaging can be used to monitor treatment change in myocardial pathology. This case sheds more light on CS and GCM being a spectrum of pathology rather than separate entities.

## Supplementary Material

There are 3 videos as supplemental materials with this case report. Descriptions are as below.
Video 1: Four-chamber SSFP imaging on presentation showing extremely poor systolic function of ventricles, mitral regurgitation, and tricuspid regurgitation.
Video 2: Four-chamber SSFP imaging in seven weeks after presentation, on Cyclosporin and Prednisone, showing improvement of both ventricular ejection fractions.
Video 3: Four-chamber SSFP imaging in nine weeks after presentation, showing relative stabilization of improvement in ejection fraction of both ventricles.
